# A Rare Coexistence of Smoldering Multiple Myeloma and JAK2-Positive Myeloproliferative Neoplasm: A Case of Dual Synchronous Hematological Malignancy

**DOI:** 10.7759/cureus.52622

**Published:** 2024-01-20

**Authors:** Adnane Hammani, Othman Doghmi, Mohammed Allaoui, Mounir Ababou, El Mehdi Mahtat, Hicham El Maaroufi, Kamal Doghmi

**Affiliations:** 1 Hematology, Mohammed V Military Training Hospital, Faculty of Medicine and Pharmacy, Mohammed V University, Rabat, MAR; 2 Hematology, Ibn Sina University Hospital Center, Faculty of Medicine and Pharmacy, Mohammed V University, Rabat, MAR; 3 Pathology, Mohammed V Military Training Hospital, Faculty of Medicine and Pharmacy, Mohammed V University, Rabat, MAR

**Keywords:** jak2 mutation, myeloproliferative neoplasm, monoclonal gammopathy, essential thrombocythemia, smoldering multiple myeloma, synchronous dual hematological malignancy

## Abstract

This article explores the rare case of an 82-year-old man diagnosed concurrently with essential thrombocythemia and smoldering multiple myeloma (SMM). The limited existing literature on individuals harboring both myeloproliferative neoplasm (MPN) and monoclonal gammopathy (MG) is of significant interest due to the distinct origins of these malignancies. The etiology of MG in MPN patients remains elusive, leading to speculation about a potential relationship or interplay between the two conditions. This unique case prompts a deeper exploration of the mechanisms underlying the coexistence of JAK2-positive MPN and SMM. It underscores the importance of tailored therapeutic strategies that carefully consider the inherent risks and potential adverse outcomes associated with these specific malignancies, thereby warranting further clinical research.

## Introduction

While existing literature acknowledges the coexistence of dual malignancies within the same patient [1], there is relatively limited documentation regarding the simultaneous occurrence of dual hematological malignancies (DHMs) [2,3], encompassing both myeloid and lymphoid hemopathies. A noteworthy aspect is the distinctive origin of these two malignancies from separate lineages within the hematopoietic ancestral tree [4]. DHMs can be classified as synchronous, manifesting within six months of the initial malignancy diagnosis, or asynchronous if they arise later [5].

Since its inclusion in the classification of monoclonal gammopathy (MG), smoldering multiple myeloma (SMM) has emerged as a significant aspect of MG [6], attracting attention in various clinical investigations.

Currently, no established strategies exist for treating or monitoring patients with myeloproliferative neoplasms (MPNs) and concurrent SMM. Additionally, the precise source of SMM in patients with MPN is not well understood, and there is uncertainty regarding whether an aberrant plasma cell condition arises from the identical hematopoietic clone as the MPN.

Numerous case reports have highlighted the occurrence of monoclonal gammopathy of undetermined significance (MGUS) or multiple myeloma (MM) in patients with MPN, with details from only a limited number of patient cohorts published [7]. Remarkably, to date, there have been no reported instances of the concurrent diagnosis of essential thrombocythemia (ET) and SMM. In this report, we present a case of synchronous concurrent SMM and ET and provide a comprehensive review of the existing literature.

## Case presentation

An 82-year-old man with a history of hypertension and diabetes was referred to our department for the management of thrombocytosis. Physical examination revealed no remarkable findings, and there was no evidence of lymphadenopathy or hepatosplenomegaly. Laboratory results indicated a platelet count of 946 g/L, hemoglobin of 12.5 g/dL, and a white blood cell count of 6.4 g/L. The patient had no systemic symptoms.

Thrombocytosis workup was initiated, initially excluding infections and iron deficiency. The platelet count was notably elevated, suggesting uncommonly high levels for secondary causes of thrombocytosis.

The patient’s chemistry panel results are shown in Table 1. Monoclonal protein was measured at 36.6 g/L. Serum immunofixation electrophoresis revealed IgG lambda gammopathy. Free light chain lambda was elevated, and kappa was normal.

**Table 1:** Laboratory results. AST = aspartate transaminase; ALT = alanine transaminase; CRP = C-reactive protein; LDH = lactate dehydrogenase

Test name	Result	Normal values
Creatinine, mg/L	8.2	6–13
Creatinine clearance, mL/minute	95	90–130
Corrected calcium, mmol/L	2.3	1–2.6
Albumin, g/L	43	40.2–47.6
Uric acid, mg/L	55	39–78
AST, U/L	34	0–35
ALT, U/L	23	0–40
Total bilirubin, mg/L	3	3–10
CRP, mg/L	2	0–5
Ferritin, ng/mL	105	11–300
M band in serum, g/L	36.6	
LDH, U/L	188	125–243
β2-microglobulin, mg/L	3.02	0.97–2.64
Serum light chain kappa, mg/I	12.84	3.3–19.4
Serum light chain lambda, mg/L	44	5.71–26.3
Kappa/lambda	0.29	0.26–1.65

**Figure 1:**
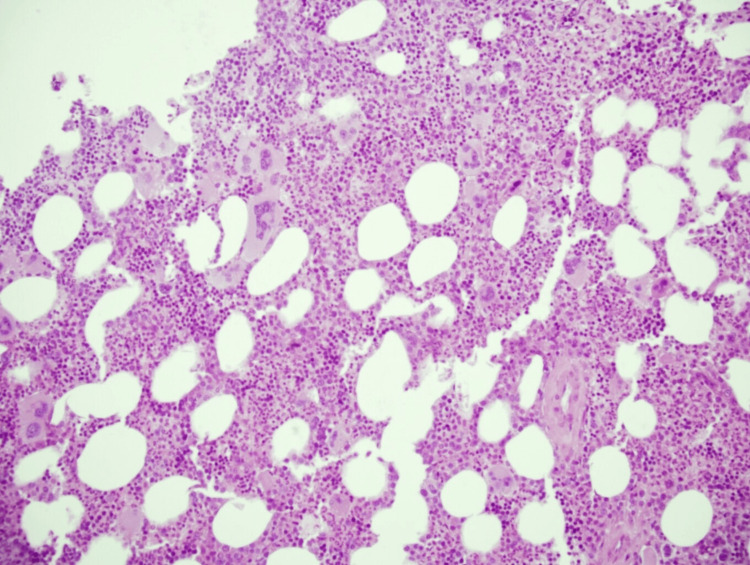
Hypercellular bone marrow biopsy, rich in lobulated megakaryocytes (hematoxylin and eosin, ×200).

**Figure 2:**
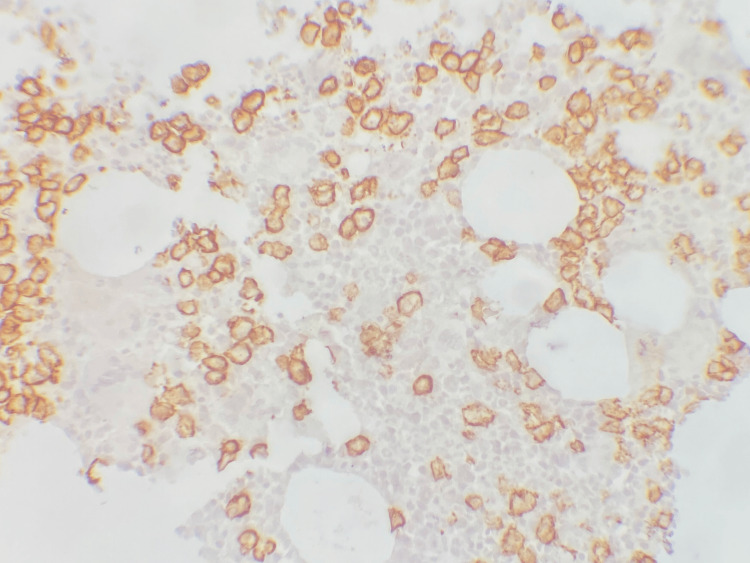
Positive immunostaining of plasma cells by CD138.

The bone marrow histological examination demonstrated hypercellularity, marked by an increased count of enlarged, mature, and hyperlobulated megakaryocytes (Figure 1). Immunostaining using the CD138 antibody revealed infiltration by plasma cells, estimated at approximately 30% (Figure 2).

The fluorescence in situ hybridization analysis revealed positivity for translocation (4,14) and a gain of 1q. Whole-body low-dose computed tomography imaging did not identify any lytic bone lesions. Molecular testing confirmed the presence of a *JAK2V617F *mutation and excluded *BCR-ABL1* gene fusion through reverse transcription polymerase chain reaction.

Based on molecular and morphological findings, the diagnosis was identified as MPN with features consistent with ET. The monoclonal protein on serum immunofixation and increased plasma cells without myeloma-defining events aligned with the diagnostic criteria indicative of a plasma cell disorder, specifically SMM. Following a comprehensive workup, the patient was diagnosed with concurrent ET and SMM.

**Figure 3:**
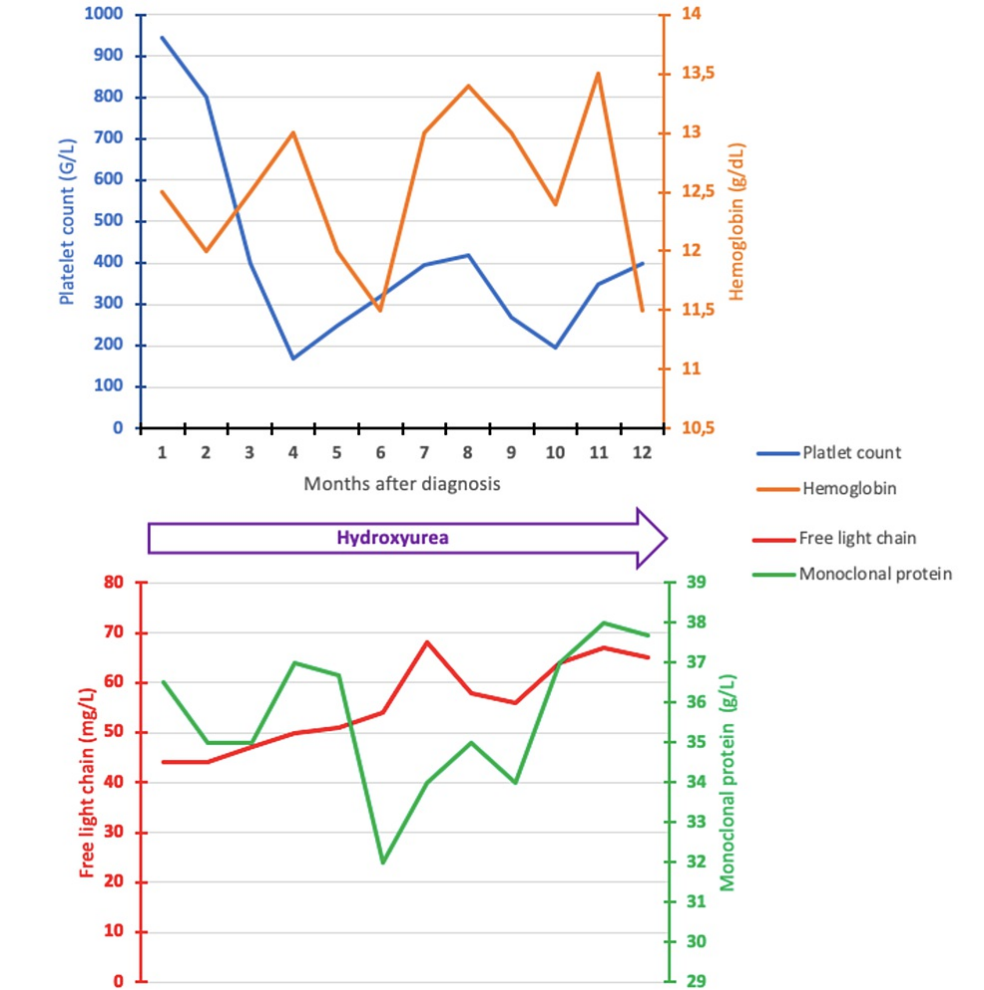
Dynamics of platelet counts, hemoglobin, monoclonal protein, and free light chain lambda during the patient management.

Treatment with aspirin and hydroxyurea was initiated, resulting in the stabilization of platelet counts (398 g/L). The patient remained free of myeloma-defining events during one year of follow-up (Figure 3).

## Discussion

This case represents one of the rarely documented reports of a patient concurrently diagnosed with ET and SMM. The limited literature on reported cases of individuals harboring both MPN and MG is of significant interest, primarily due to the distinct origins of these malignancies from two separate cellular lineages in hematopoiesis.

The etiology of MG in patients with MPN remains elusive, displaying potential variability among individuals. A plausible hypothesis revolves around the inflammatory condition and heightened cytokine levels linked to MPN, fostering the proliferation of existing monoclonal plasma cells [7].

An alternative possibility suggests that a shared hematopoietic precursor gives rise to both the MPN and a clonal plasma cell population [7]. Nevertheless, in a case report illustrating the coexistence of MM and ET, the origin of the two diseases was demonstrated to be from distinct clones, each with independent genetic mutations [8].

To date, no documented cases of SMM associated with ET at the diagnostic stage have been reported. Nevertheless, instances of MG, other than asymptomatic myeloma (MGUS and MM), have been detailed (Table 2).

**Table 2:** Report of cases of ET with concomitant MG. HU = hydroxyurea; PC = plasma cells; NR = not reported; MG = monoclonal gammopathy; ET = essential thrombocythemia; MPN = myeloproliferative neoplasms; MGUS = monoclonal gammopathy of undetermined significance; MM = multiple myeloma

Patient	Sex	Age (year)	Duration from ET to MG	MPN mutation	MPN treatment	PC disorder	References
1	Female	77	0 months	JAK2 V617F	HU	IgG L MGUS	Javorniczky et al., 2020 [7]
2	Female	71	1 year	CALR	HU	IgM K MGUS	Javorniczky et al., 2020 [7]
3	Male	68	4 year	JAK2 V617F	HU	IgG K MGUS	Javorniczky et al., 2020 [7]
4	Female	67	0 months	JAK2 V617F	HU	IgG L MM	Kuroda et al., 2009 [8]
5	Male	71	4 years	JAK2 V617F	-	IgG K MM	Badelita et al., 2015 [9]
6	Male	68	2 years	JAK2 V617F	HU	IgA L MM	Terzi et al., 2015 [10]
7	Male	66	6 years	JAK2 V617F	HU	IgG K MM	Youssef et al., 2013 [11]
8	Female	32	1 month	JAK2 V617F	NR	IgG L MM	Naeem et al., 2019 [12]

When managing patients in this context, it is crucial to prioritize the treatment of malignancies demonstrating heightened aggressiveness or the possibility of transformation to acute leukemia. The therapeutic strategy should be adapted to address these specific malignancies, considering their inherent risks and potential for adverse outcomes. In our case, the patient underwent cytoreductive therapy for ET and a surveillance protocol for SMM. The surveillance involved regular monitoring and observation to track disease progression or the emergence of symptoms, facilitating timely intervention if necessary.

Limitations included the scarcity of existing literature on this particular case due to its novelty, posing a challenge in extrapolating findings and generalizing conclusions.

## Conclusions

In the context of previously acknowledged associations between MPN and MG, this case stands out as a unique instance among published reports, highlighting the simultaneous presence of JAK2-positive MPN and SMM. The exact mechanism underlying this occurrence remains a subject of debate, especially considering the distinct lineages involved. Ongoing clinical investigation and analysis are crucial for a deeper understanding of the clonal origin, prognosis, and the optimal therapeutic approach for managing these coexisting conditions.
